# A Case of Multi-Drug Resistant Tubercular Pyomyositis in an Immunosuppressed Patient Presenting as Bacterial Infection

**DOI:** 10.31138/mjr.090823.adm

**Published:** 2023-08-09

**Authors:** Ujjwol Risal, Suravi Pandey, Pradeep Raj Regmi, Anup Subedee

**Affiliations:** 1 Department of Internal Medicine, Hospital for Advanced Medicine and Surgery, Dhumbarahi, Kathmandu, Nepal,; 2 Department of Radiology, Hospital for Advanced Medicine and Surgery, Dhumbarahi, Kathmandu, Nepal,; 3 Department of Internal Medicine, Kirtipur Hospital, Kirtipur, Kathmandu, Nepal

**Keywords:** pyomyositis, immunosuppressed, MDR TB

## Abstract

Tubercular pyomyositis is a rare but distinct clinical entity which is difficult to diagnose especially in a patient with underlying autoimmune disease. The treatment is even more challenging if it is a multi-drug resistant strain. Here we report a patient with primary Sjögren’s syndrome who presented with persistent inflammation of his right arm which was later diagnosed as multi-drug resistant tubercular pyomyositis. This case highlights the need for a high index of suspicion for tuberculosis in all cases of pyomyositis.

## INTRODUCTION

Tubercular pyomyositis is a rare clinical entity which is difficult to diagnose because of its resemblance to other infectious and inflammatory causes of myositis. The diagnosis is even more challenging in a patient with underlying autoimmune disease on immunosuppressive drugs. We report a patient with primary Sjögren’s syndrome who presented with persistent right arm inflammation which was later proven to be multi-drug resistant tuberculosis (TB).

## CASE PRESENTATION

**Figure 1.**
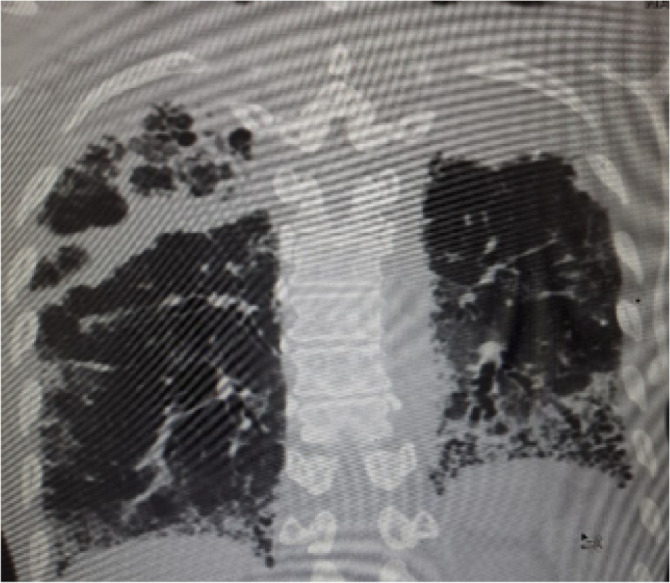
HRCT of chest (Coronal section) showing extensive bilateral basal honeycombing and traction bronchiectasis.

**Figure 2.**
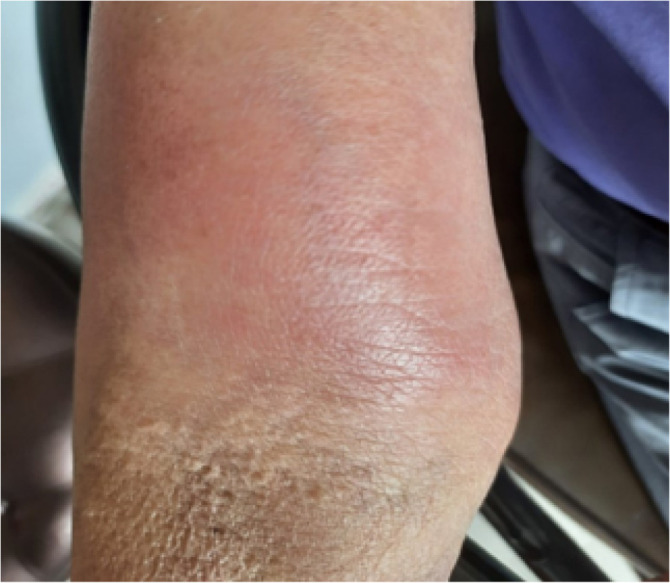
Red, shiny, and swollen anterior aspect of right distal arm.

An eighty-two-year-old male of Brahmin ethnicity from Kathmandu Nepal, presented to us with swelling, redness, and warmth of right arm of two days’ duration. About two months prior to this presentation, he was diagnosed with primary Sjögren’s syndrome (PSS) based on his five-year-long history of xerostomia and keratoconjunctivitis sicca, recently diagnosed interstitial lung disease (usual interstitial pneumonia pattern) (**Figure 1**), constitutional symptoms of fatigue and weight loss, positive Schirmer’s test, positive anti-nuclear antibody (3+speckled), SSA and SSB antibodies. He had also been diagnosed as a case of pemphigus foliaceous five months prior to presentation and was being managed by a dermatologist. His past history included hypertension. He was a non-smoker and a social drinker. His medications included azathioprine 50 mg, prednisolone 15 mg (on taper) and losartan 50 mg. Physical examination of right arm showed tender, red, and warm anterior aspect confined to the distal arm (**Figure 2**). Bilateral infra-scapular Velcro crepitations were heard on respiratory system examination. The rest of the systemic examination was normal. Ultrasound of the right arm showed features suggestive of cellulitis with underlying myositis. A working diagnosis of cellulitis was made, and he was sent home on amoxicillin-clavulanate and anti-inflammatory medications. He presented a couple of days later with fever, increased swelling and redness of right arm. His laboratory tests showed elevated inflammatory markers, deranged renal function, and anaemia (**Table 1**). He was admitted with intravenous cefepime and linezolid with the working diagnosis of right arm cellulitis with acute kidney injury. After a few of days of antibiotics, his swelling and redness subsided, his systemic symptoms improved, and his inflammatory markers showed a falling trend (**Table 1**). He was finally discharged after one week of antibiotics.

**Table 1.** Laboratory investigations.

	**At first admission**	**At the time of discharge**	**At second admission**	**At the time of discharge**
**Hb (gm/dl)**	7	10.6	10.5	9.5
**TC (/mm^3^)**	6590	9410	12720	6950
**DC**	N62L24E2M12	N70L20E01M09	N85L08E01M06	N72L19E02M)7
**Platelet (/mm^3^)**	391000	3400000	159000	194000
**Urea (mg/dl)**	52	50	57	
**Creatinine (mg/dl)**	1.7	1.3	1.1	
**ESR (mm/hr)**	51			
**CRP (mg/L)**	103.45	13.52	17.7	28.61

**Figure 3.**
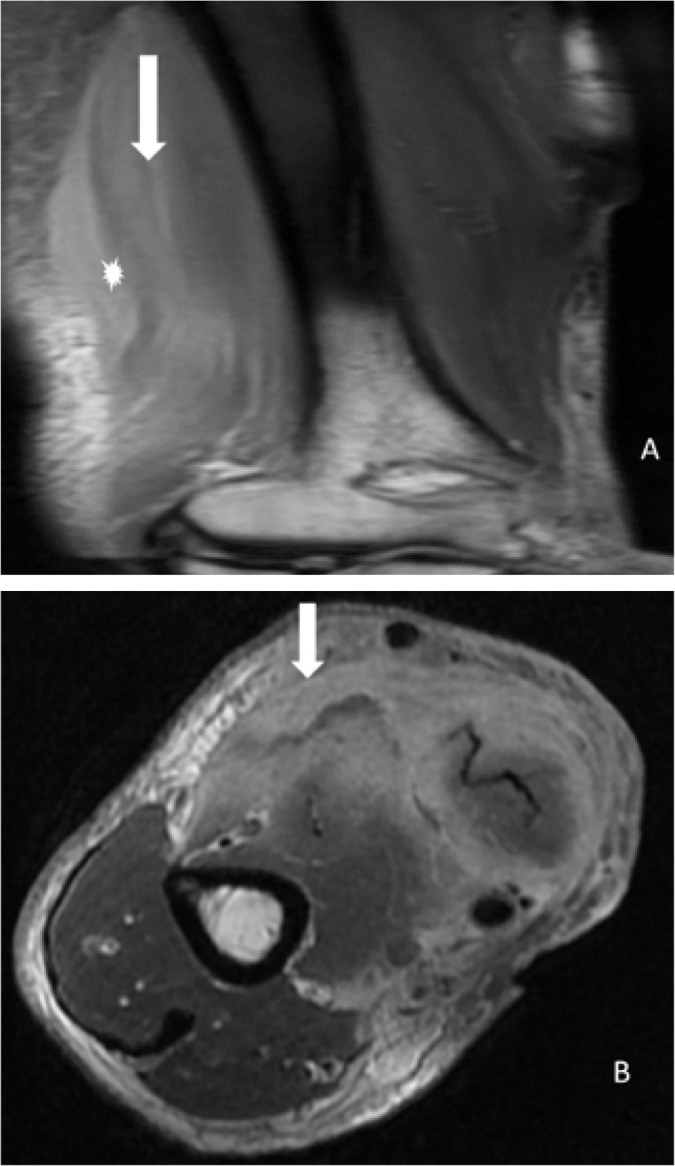
(A) Sagittal PD FS MR Image shows ill-defined T2 high signal intensity within the muscles of the arm anterior to the cortex of humerus (shown by white arrow) along with collection (shown by asterisk). (B) Axial PD FS MR Image shows diffuse high signal intensity within the muscles of right arm (shown by white arrow), suggesting inflammation.

**Figure 4.**
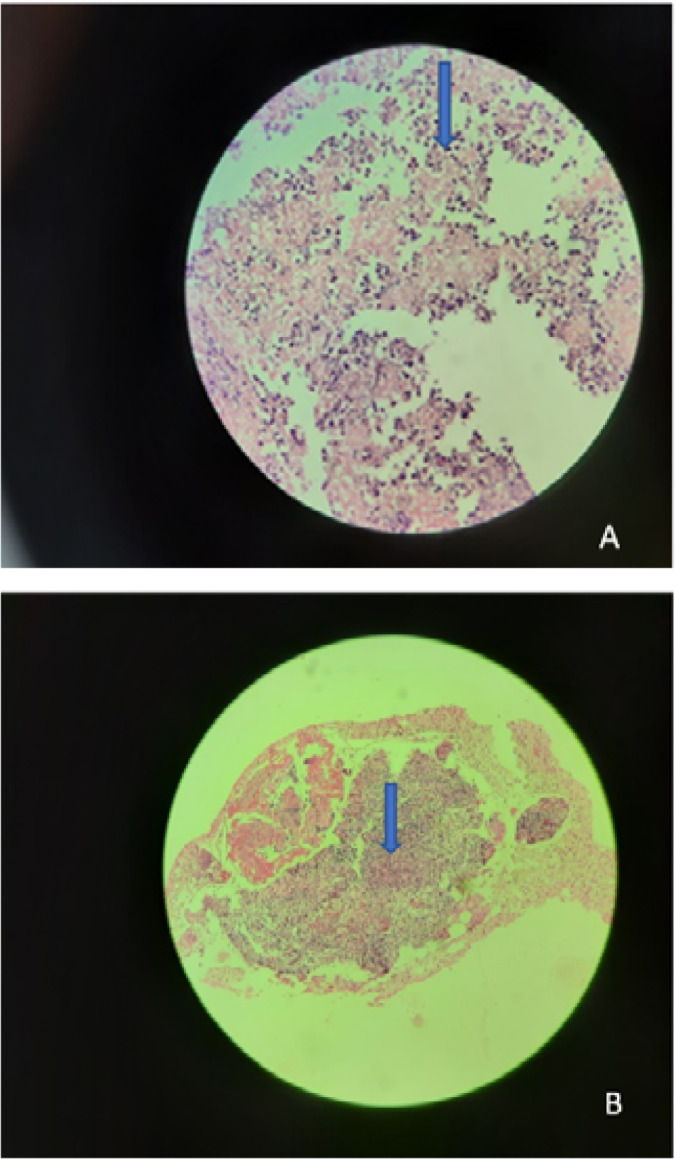
(A) Muscle biopsy showing caseous necrosis (arrow). (B) Muscle biopsy showing epithelioid cell granuloma (arrow).

He presented ten days later with similar symptoms of fever, pain, redness, and warmth of the same site. He was admitted again and started on intravenous linezolid since he had responded well to it in the prior admission. He underwent a magnetic resonance imaging (MRI) of his right arm which showed mild fluid collection with muscle inflammation (**Figure 3**). Again, after a couple of days of linezolid, inflammation in his arm subsided and his blood parameters improved. This time he was planned for a longer duration of antibiotics to cover any residual infection. He was discharged on oral linezolid after five days’ admission. At follow-up in seven days, his symptoms had resolved but there was still some redness and oedema. Therefore, linezolid was continued, which was planned for at least another week or so. Meanwhile the patient wanted to go to another centre for a second opinion. During his work-up at another centre, which also included a biopsy of his right arm muscle, it was concluded that the persistent inflammation was secondary to inflammatory myositis. His prednisolone dose was increased to 30 mg once daily to be tapered by 5 mg every week. When he came back to us after a week’s treatment, he complained of pus discharge from his biopsy site for two days. On examination there was soakage of his dressing. Incision and drainage of the site was done. The pus was sent for analysis and a repeat biopsy was taken. The Gene Xpert of the pus was positive for *Mycobacterium tuberculosis* which also showed resistance to rifampicin. The repeat biopsy showed caseous necrosis with multiple granulomas (**Figure 4**). The Ziehl-Neelsen stain of the pus was positive for acid fast bacilli. A final diagnosis of rifampicin resistant tubercular pyomyositis was made and the patient was referred to the local tuberculosis (TB) treatment centre for starting anti-tubercular therapy. He was started on linezolid, pyrazinamide, levofloxacin, bedaquiline, and clofazimine. After two months of therapy, his pain and swelling subsided and his constitutional symptoms improved. At the time of writing this manuscript, his pus culture came back positive for *Mycobacterium tuberculosis*. Drug sensitivity testing showed resistance to both isoniazid and rifampicin.

## DISCUSSION

Pyomyositis is a primary bacterial infection of the skeletal muscles. It is also known as tropical pyomyositis since it is more common in the tropics.^1^ It is particularly common among immunosuppressed individuals.^2^ The most common organisms causing pyomyositis include *Staphylococcus aureus*, Group A *streptococcus* and less commonly Group B, C and G *Streptococcus*, *pneumococcus*, Haemophilus spp., and Gram-negative bacilli.^3^ *Mycobacterial tuberculosis* has also been reported to cause pyomyositis but is extremely rare.^4^

Bone and joint TB currently account for 15–20% of all extra-pulmonary (EPTB) cases in developing countries.^5^ It is the third most common type of EPTB after lymphatic and pleural TB.^6^ Vertebral or spinal TB is the most common form of musculoskeletal TB (MSK TB) accounting for almost half of all bone and joint TB.^7^ Tubercular myositis is an uncommon presentation of MSK TB and is usually the result of contiguous spread from bone or joint infections. It is commonly seen as psoas abscess in Pott’s spine.^6^ Primary myositis is a rare manifestation of MSK TB and is seen more commonly seen in immunocompromised patients.^4^ Our patient also had many risk factors for developing primary myositis; ie, old age, steroids (about 3 gm cumulative dose of prednisolone over two months) and azathioprine use, residence in a region with high TB endemicity, and his primary disease itself which was an independent risk factor for the development of TB.^8^ The pathogenesis of primary myositis is currently unknown but is hypothesised to occur following hematogenous spread from the primary focus.^6^ However the probability of finding active pulmonary TB on a chest X-ray is only 29% in these cases.^9^ We could not find any local or distant focus of infection in our patient. Even though the typical clinical features of frank pyomyositis and constitutional features like fever, weight loss, and anorexia are reported to be less common in TB pyomyositis, our patient had all the features of inflammation and constitutional symptoms. Imaging modalities such as ultrasound and MRI can show myositis and the extent of tissue involvement but cannot give enough information about the etiology.^6^ Our patient also had features of myositis on ultrasound and MRI but suscpicion for TB was low due to initial response to empirical antibiotics. A definitive diagnosis can only be made based on histological analysis and TB culture which were both positive in our patient. One of the factors that led to a delay in diagnosis of TB was low suspicion for TB and a partial response of the lesion to linezolid which itself is a second line anti-tuber-cular drug. In spite of the final diagnosis of TB, there were many challenges in the management of our patient which were his age, underlying autoimmune disease, and most importantly his multi-drug resistant (MDR) TB status. MDR TB is emerging as a public health threat. The World Health Organisation estimates that 3.3% of new cases occurring worldwide in 2019 had MDR/rifampicin-resistant (RR)-TB.^10^ The burden of TB in Nepal is increasing according to a survey done in 2018–19. It showed the annual incidence of 245 and prevalence of 416 per 100000 population.^11^ In a survey done in Nepal in 2011/12, the proportion of MDR-TB was 2.2% among new cases and 15.4% among retreatment cases.^11^ Our patient did not have history of TB in the past nor had he been in close contact with MDR-TB patients in the recent past. Therefore, he probably acquired the drug resistant strain from the community itself which in itself is a frightening situation. It is worrisome that even after years of research and achievements in the diagnosis and treatment of TB, it still lingers on as an unwelcome guest.

## CONCLUSIONS

Primary tubercular pyomyositis is a rare entity but should be suspected in cases of localised myositis which does not respond to antibiotics especially in immunocompromised patients. The possibility of MDR TB should never be ruled out in any case of TB.
